# Supplementary figures and images for: Cobalamin Riboswitches Are Broadly Sensitive to Corrinoid Cofactors to Enable an Efficient Gene Regulatory Strategy
Source: mBio. 2022 Aug 22;13(5):e01121-22. doi: 10.1128/mbio.01121-22 (PMC9600662; doi:10.1128/mbio.01121-22)

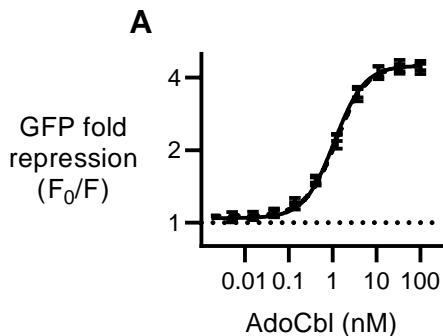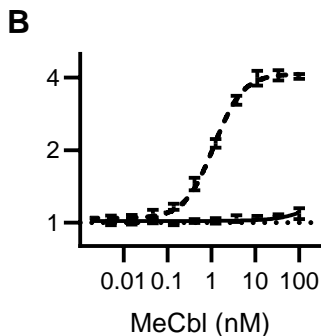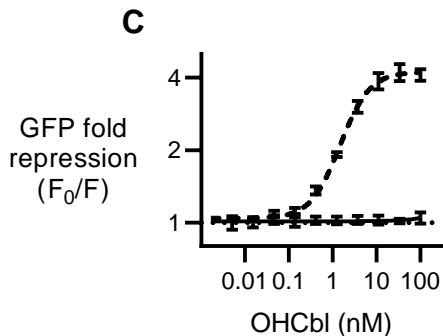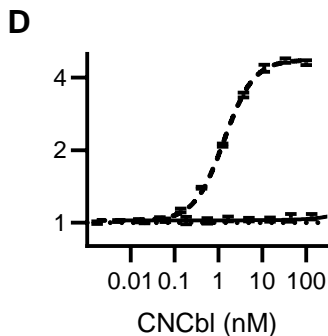

--- adenosyltransferase expressed  
— adenosyltransferase deleted ( $\Delta btuR$ )

Supplement: FIG S1 [file mbio.01121-22-sf001.pdf]

A

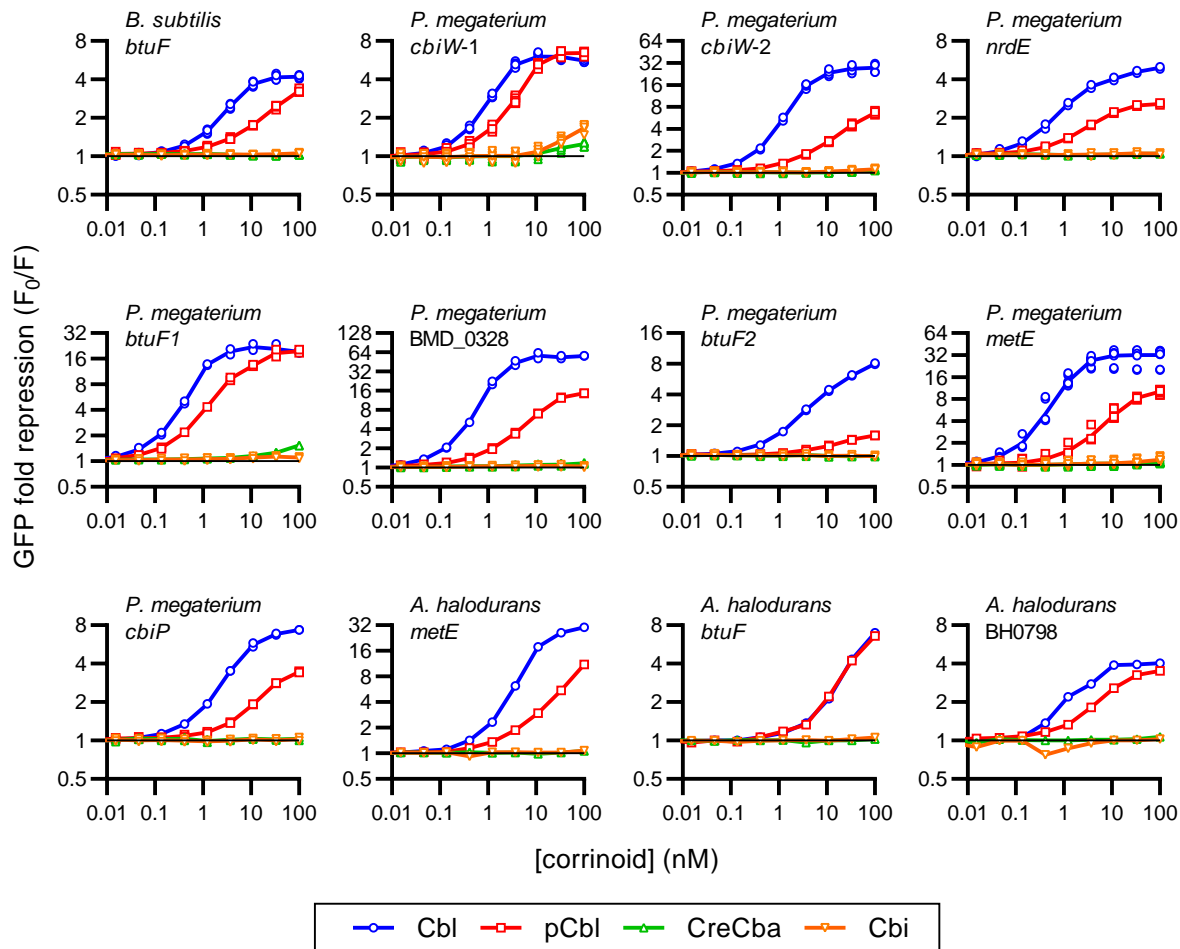

**B**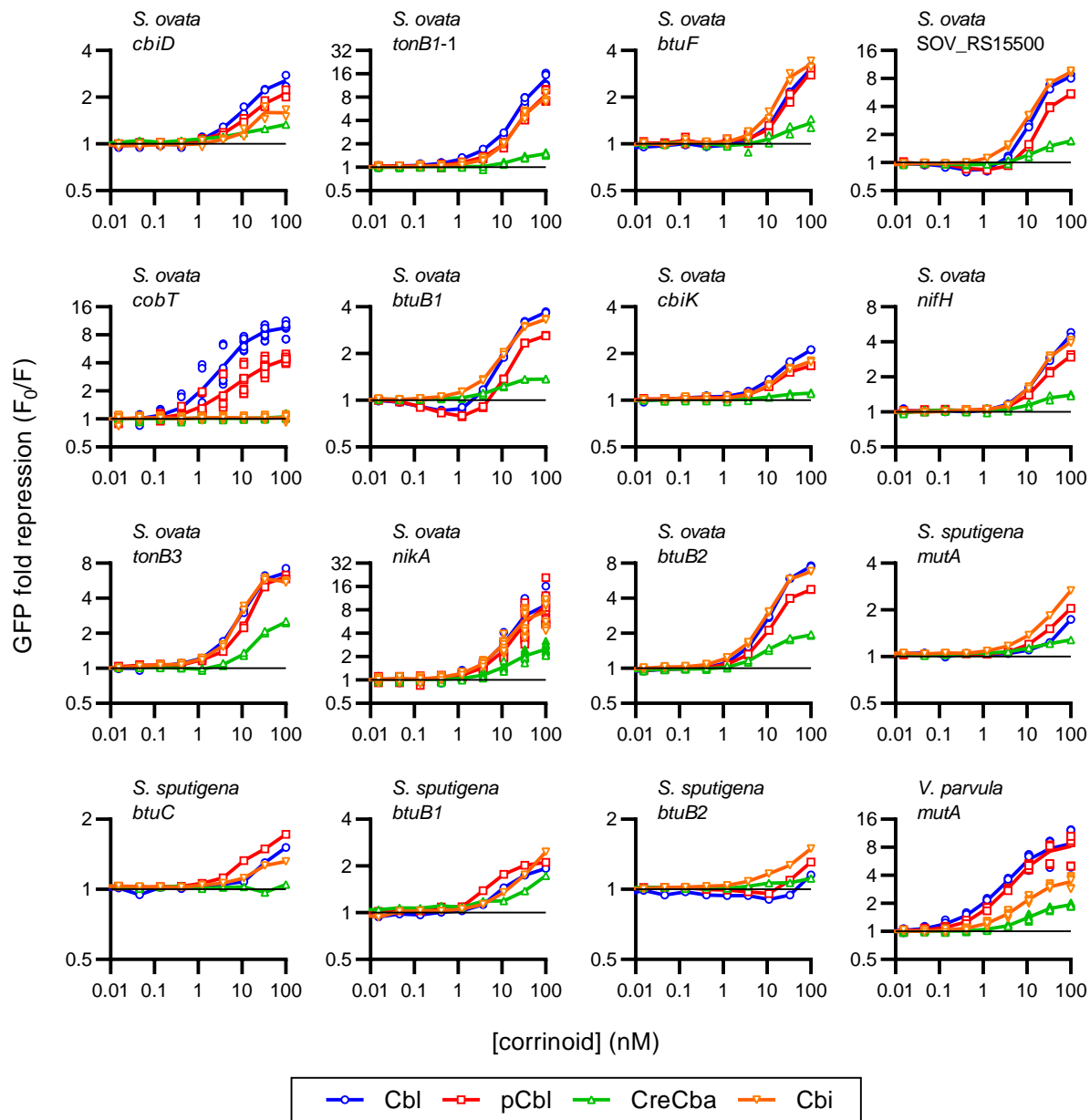

C

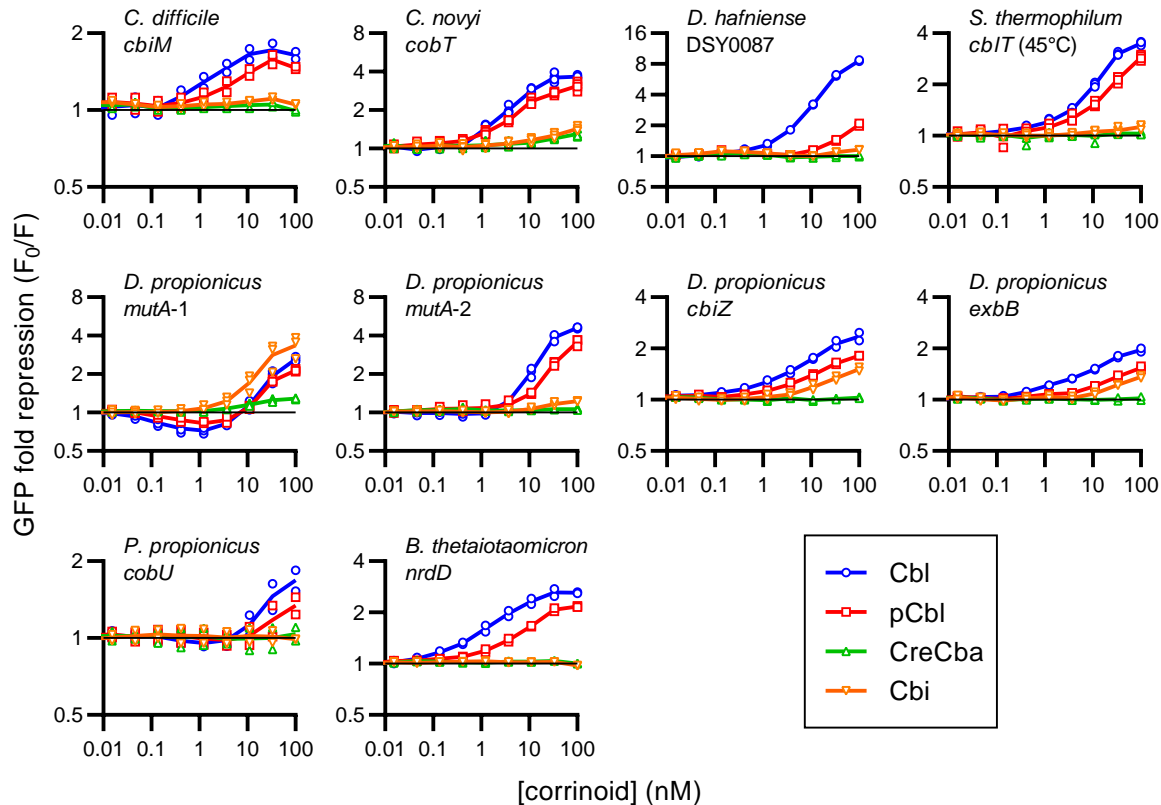

Supplement: FIG S2 [file mbio.01121-22-sf002.pdf]

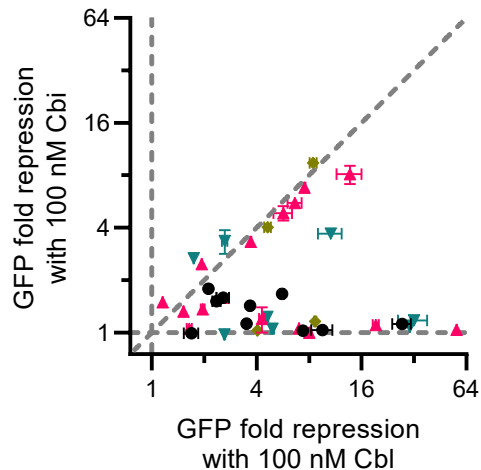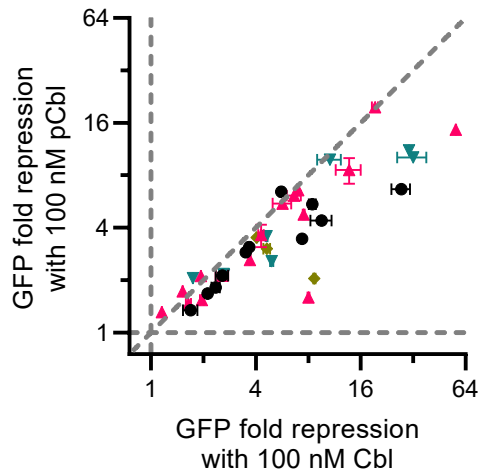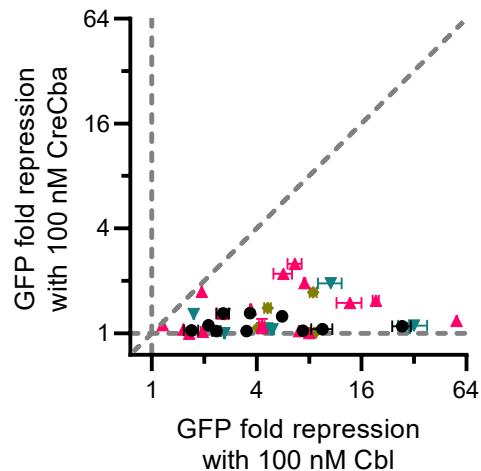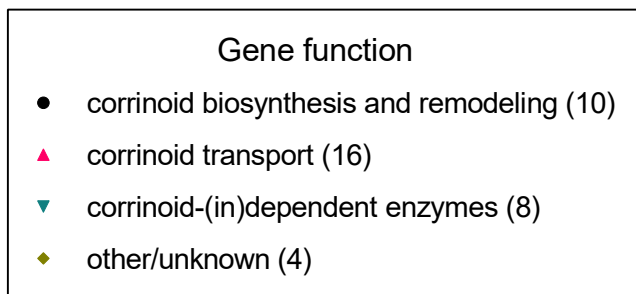

Supplement: FIG S3 [file mbio.01121-22-sf003.pdf]

**A**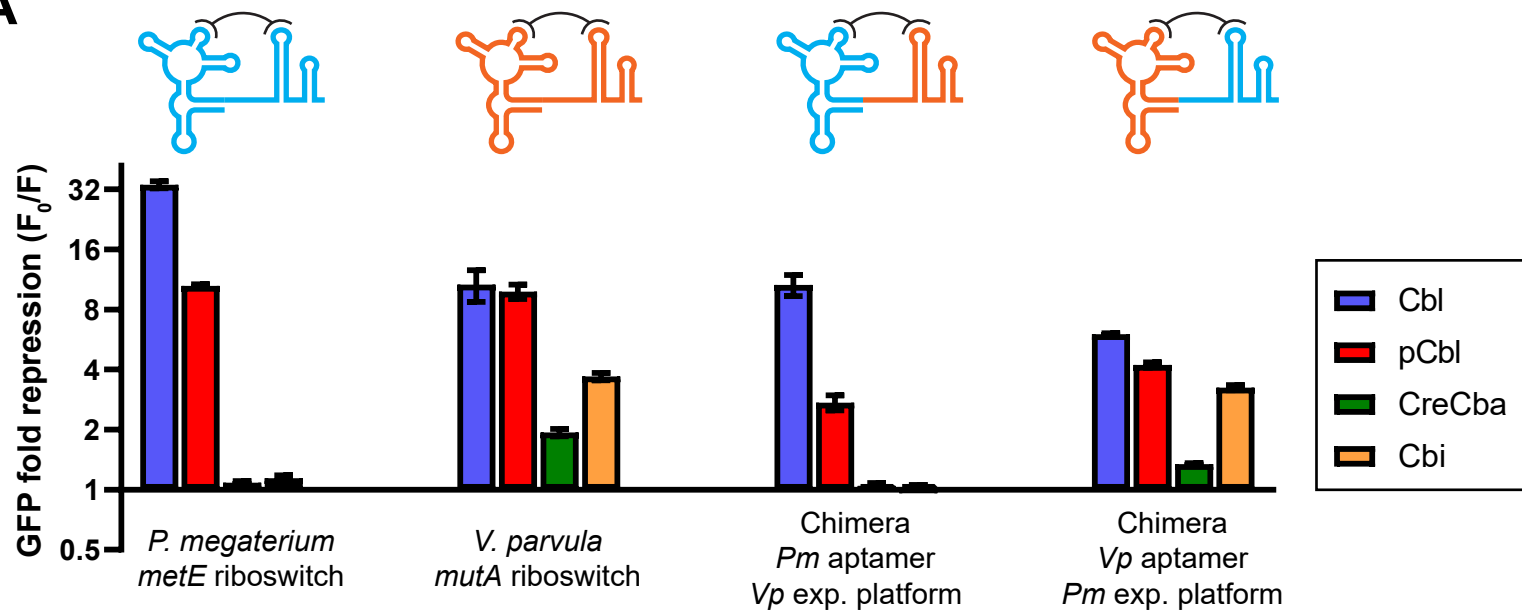**B**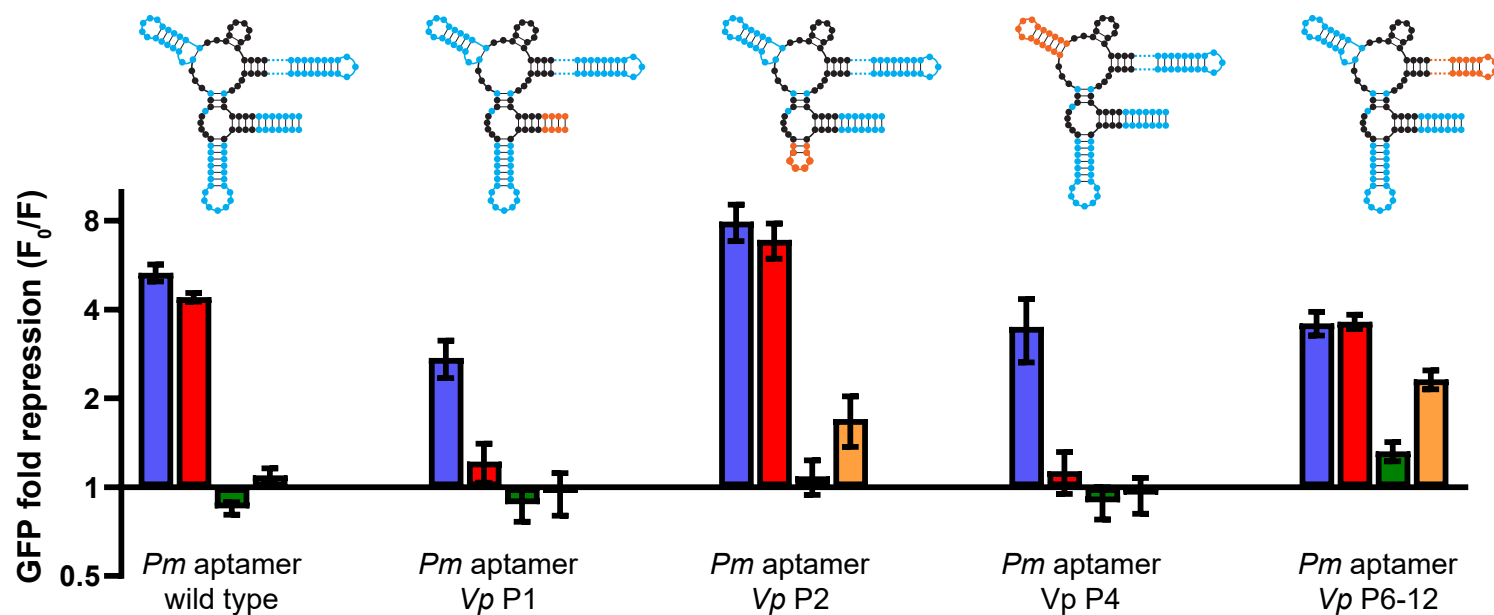**C**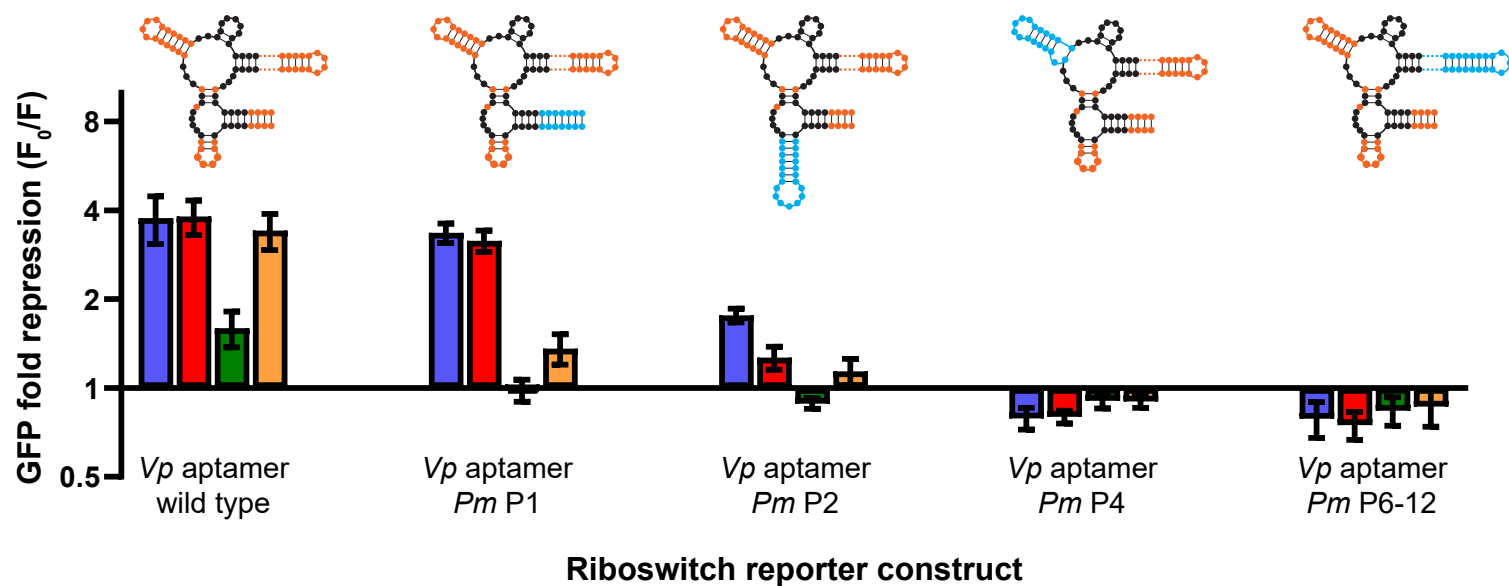

Supplement: FIG S4 [file mbio.01121-22-sf004.pdf]

Relative absorbance (Abs / Abs<sub>264 nm</sub>)

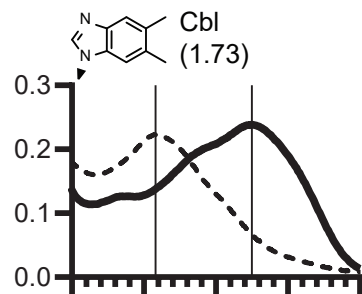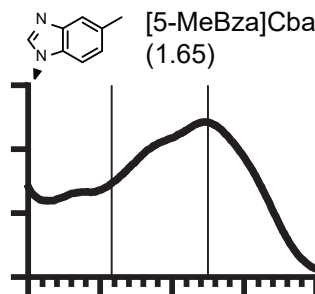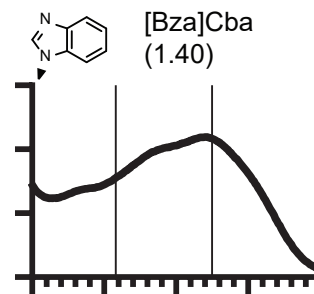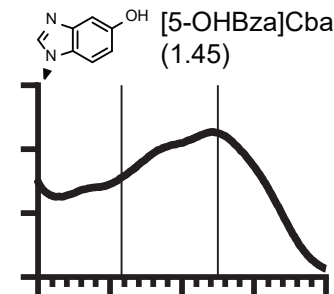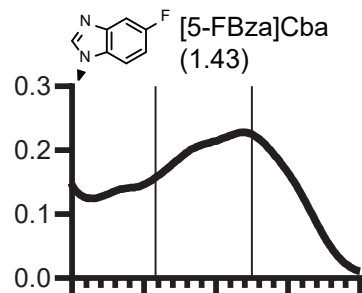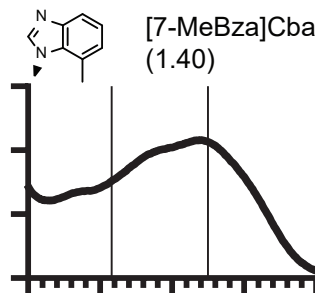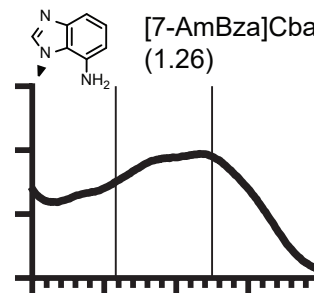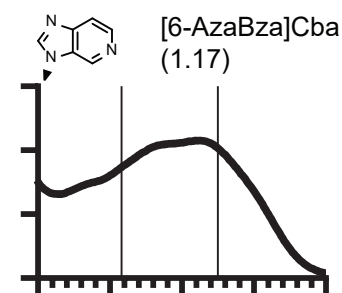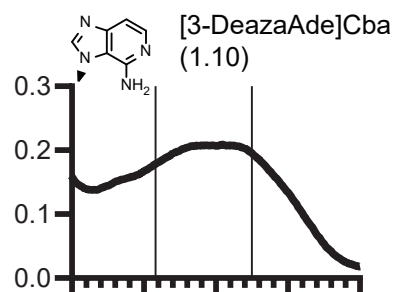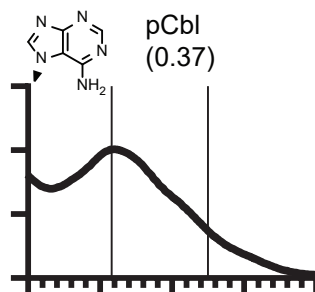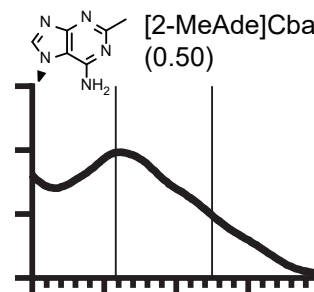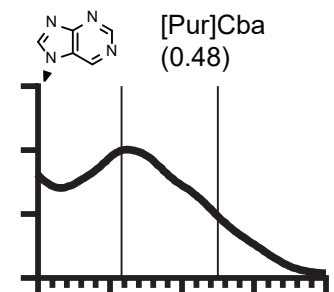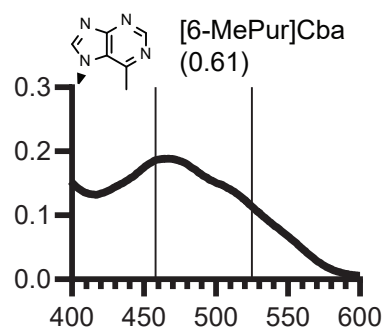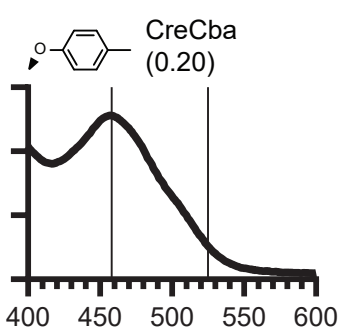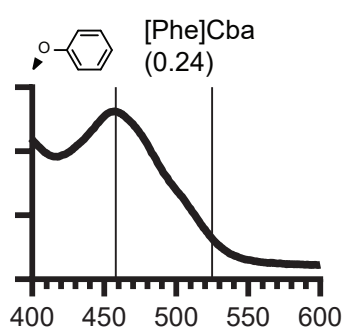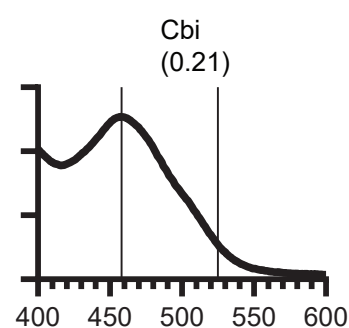

Wavelength (nm)

Supplement: FIG S5 [file mbio.01121-22-sf005.pdf]

**A**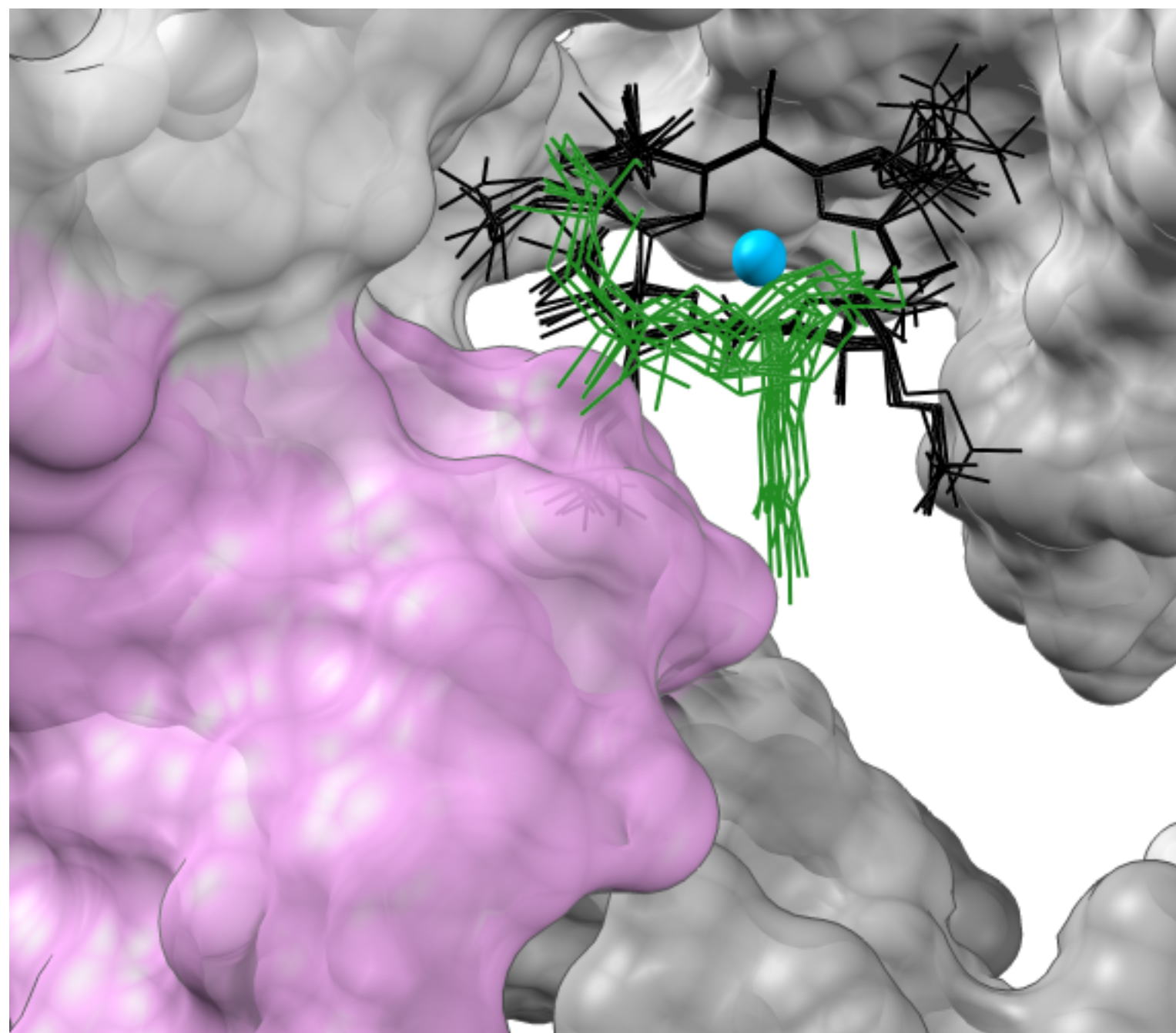**C**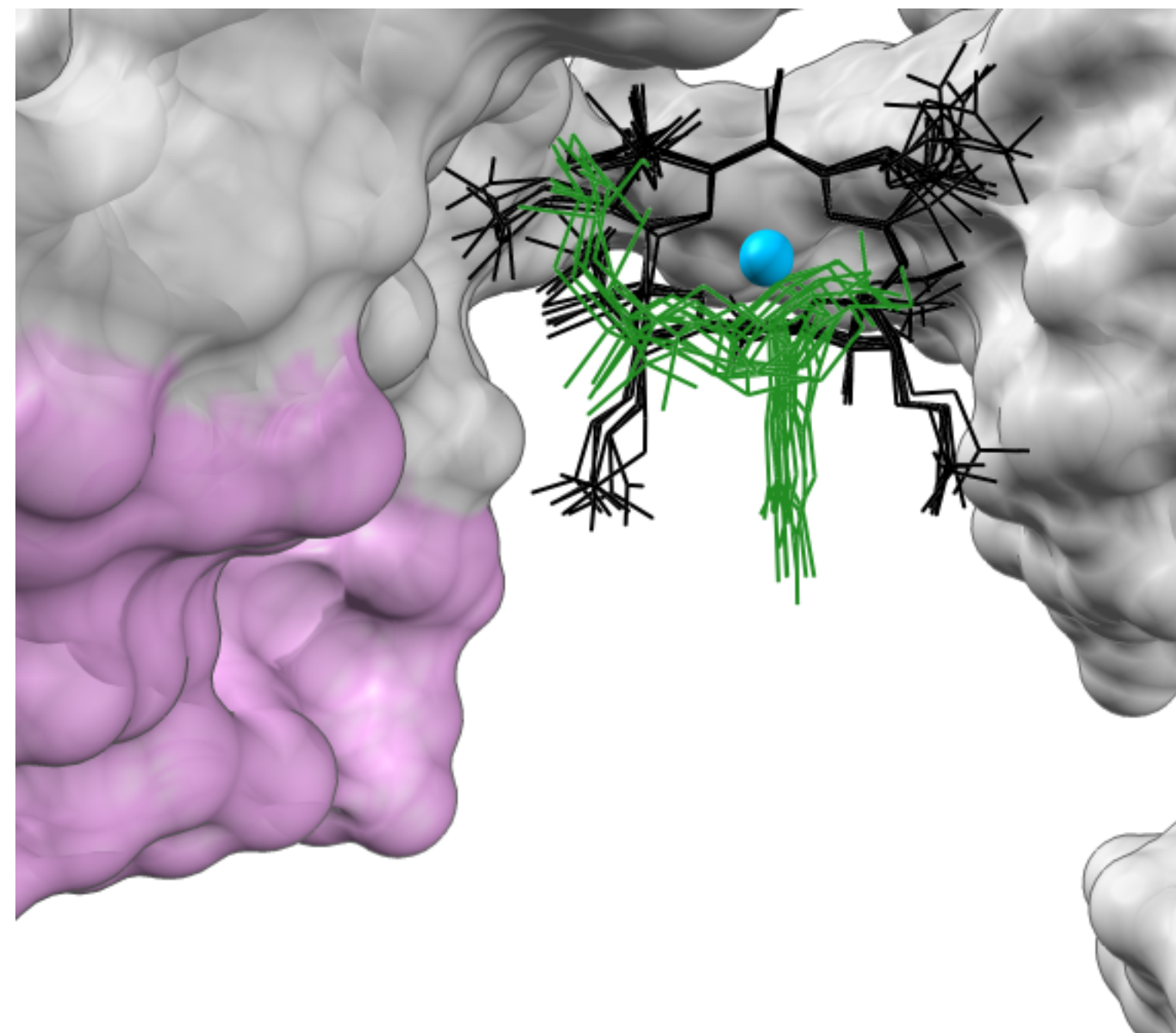**E**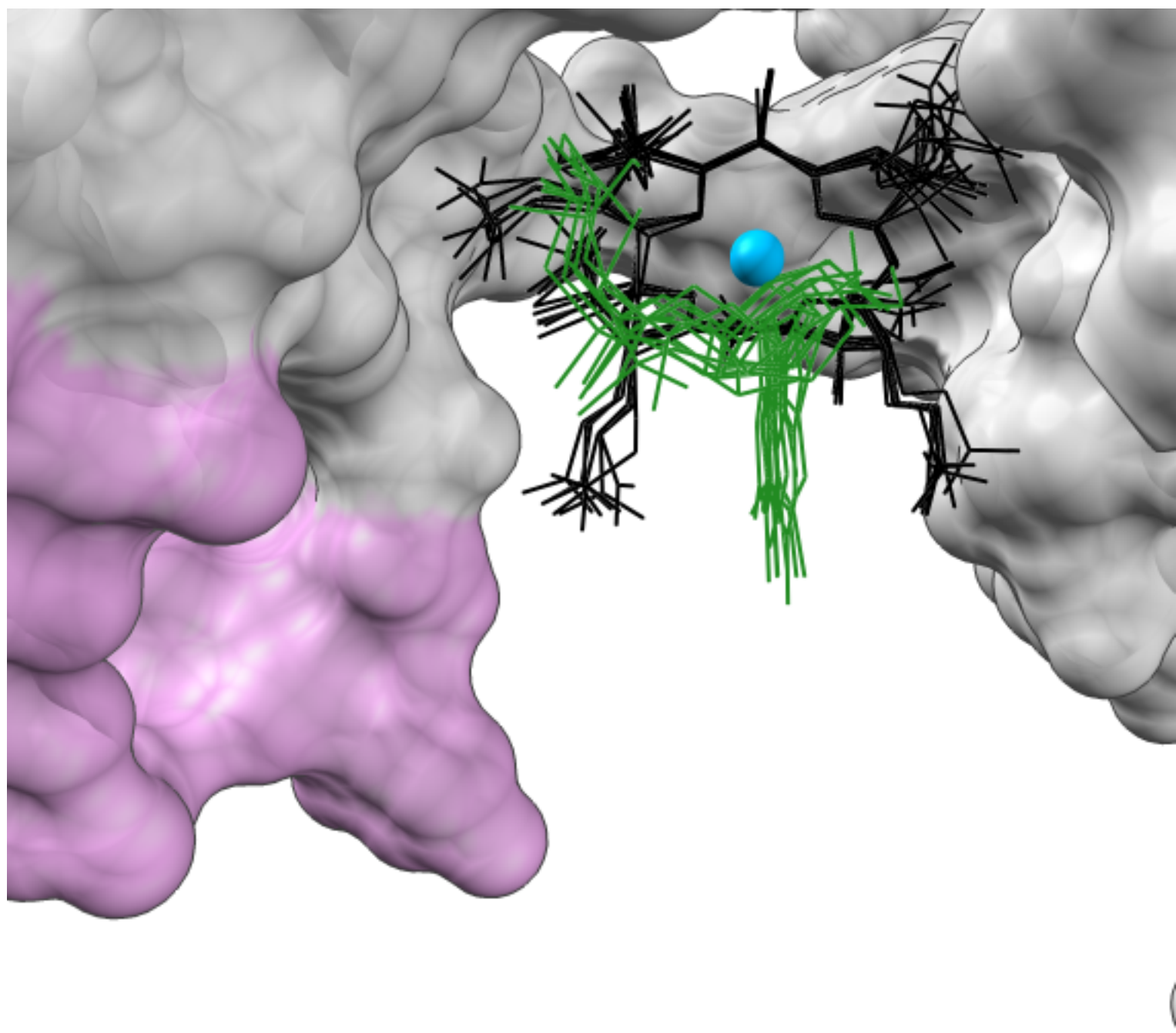**B**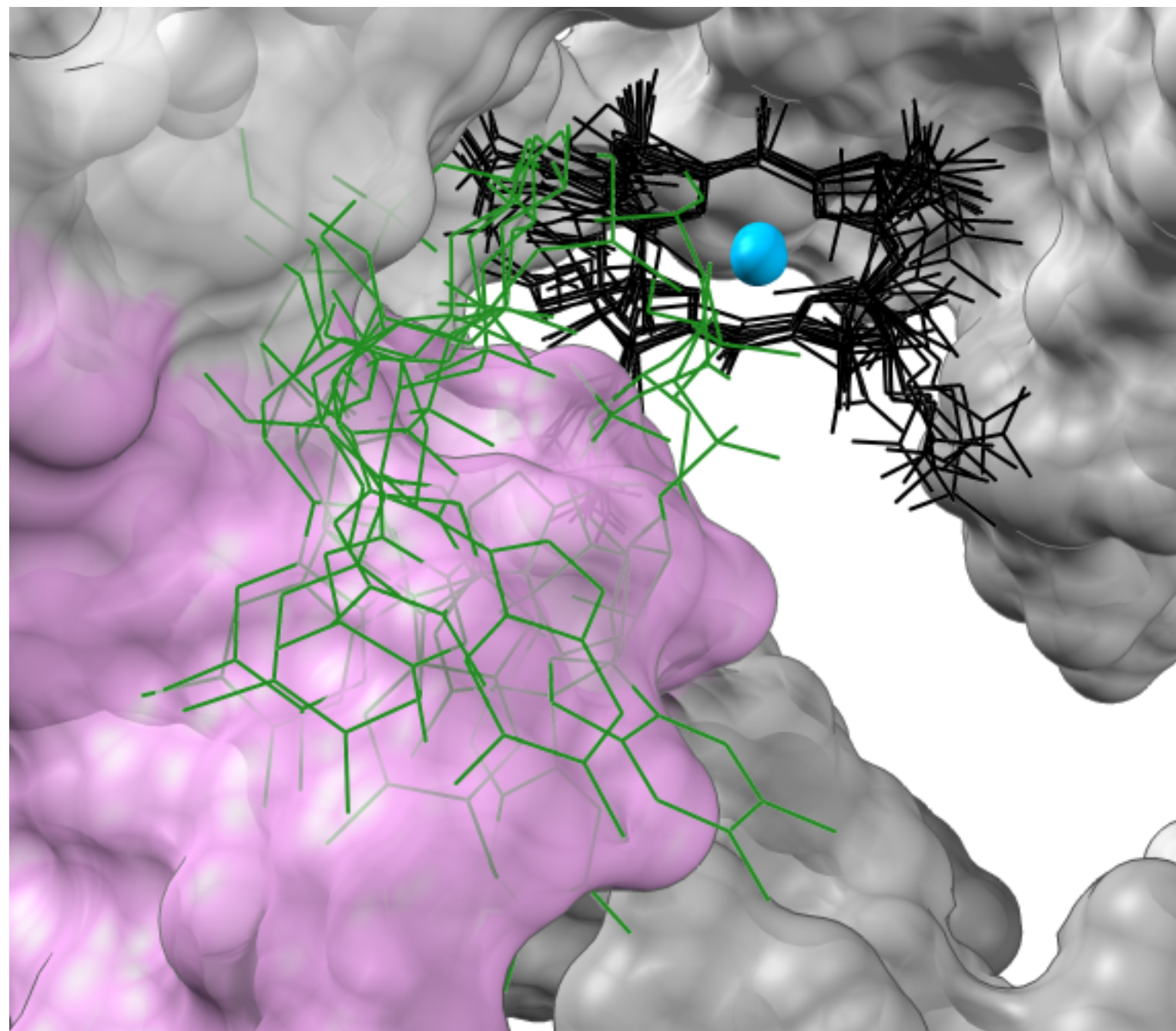**D**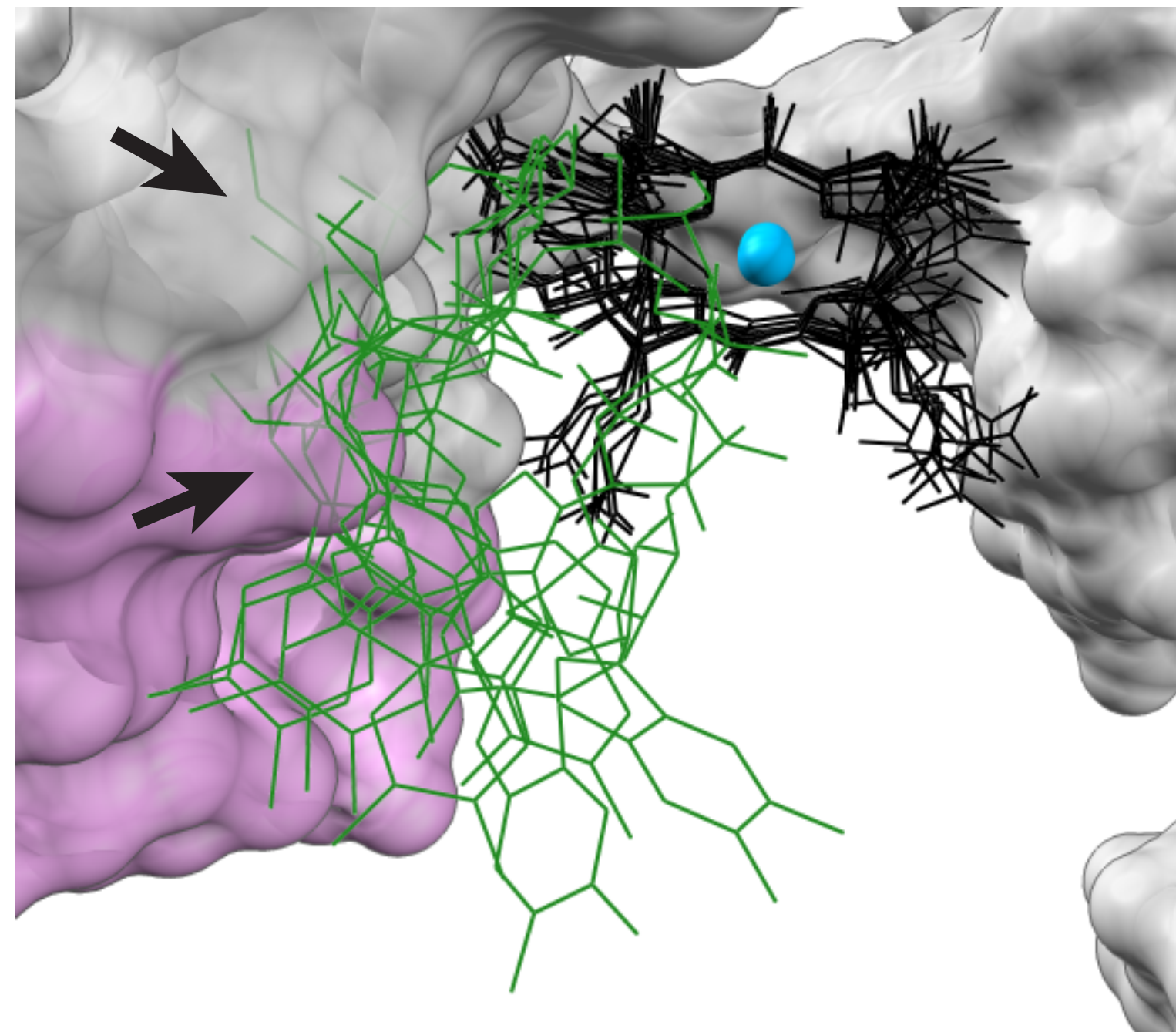**F**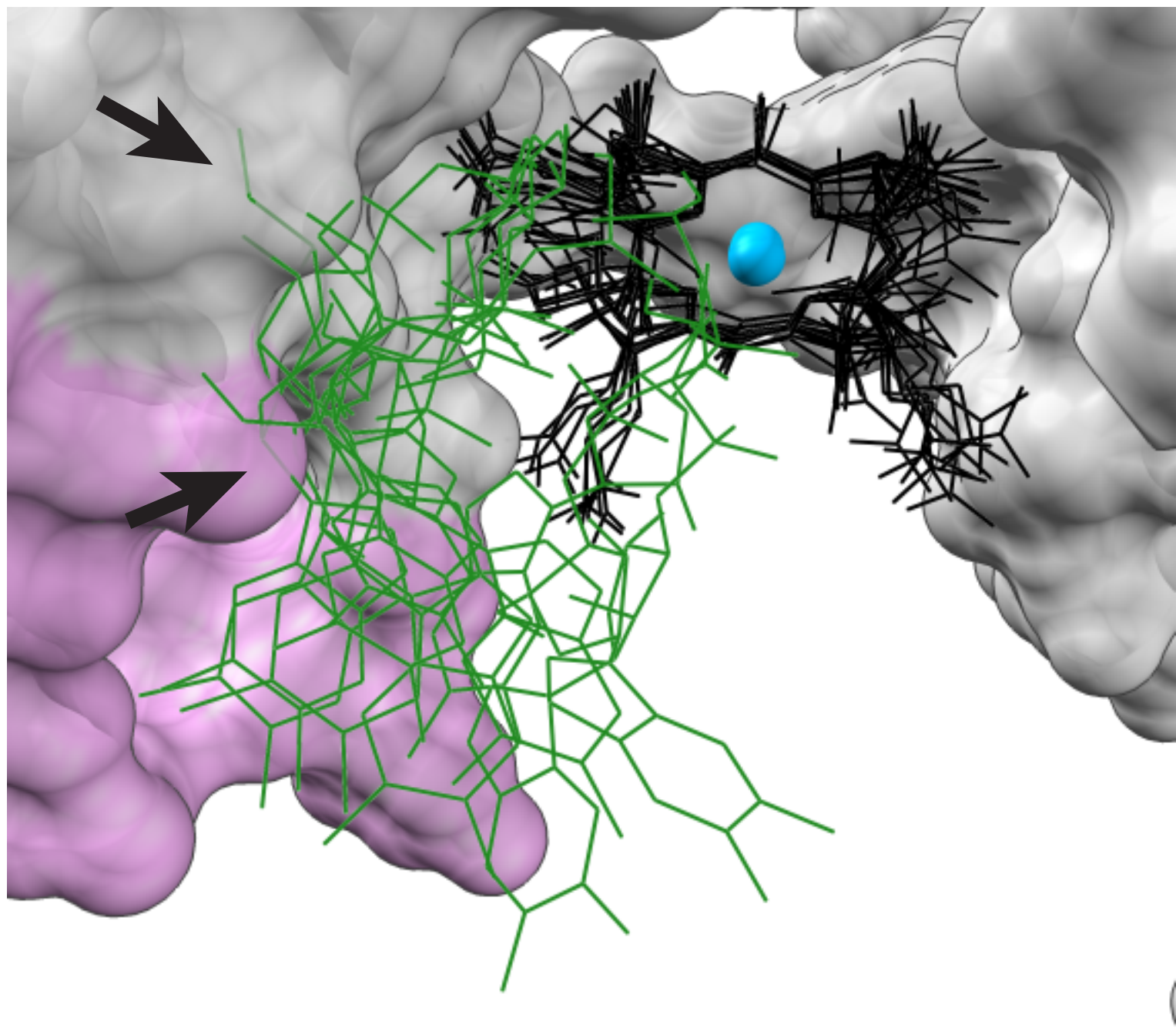

Supplement: FIG S6 [file mbio.01121-22-sf006.pdf]
