# Supplementary material for: Cobalamin Riboswitches Are Broadly Sensitive to Corrinoid Cofactors to Enable an Efficient Gene Regulatory Strategy
Source: mBio. 2022 Aug 22;13(5):e01121-22. doi: 10.1128/mbio.01121-22 (PMC9600662; doi:10.1128/mbio.01121-22)
Supplement: FIG S7 [file mbio.01121-22-sf007.pdf]

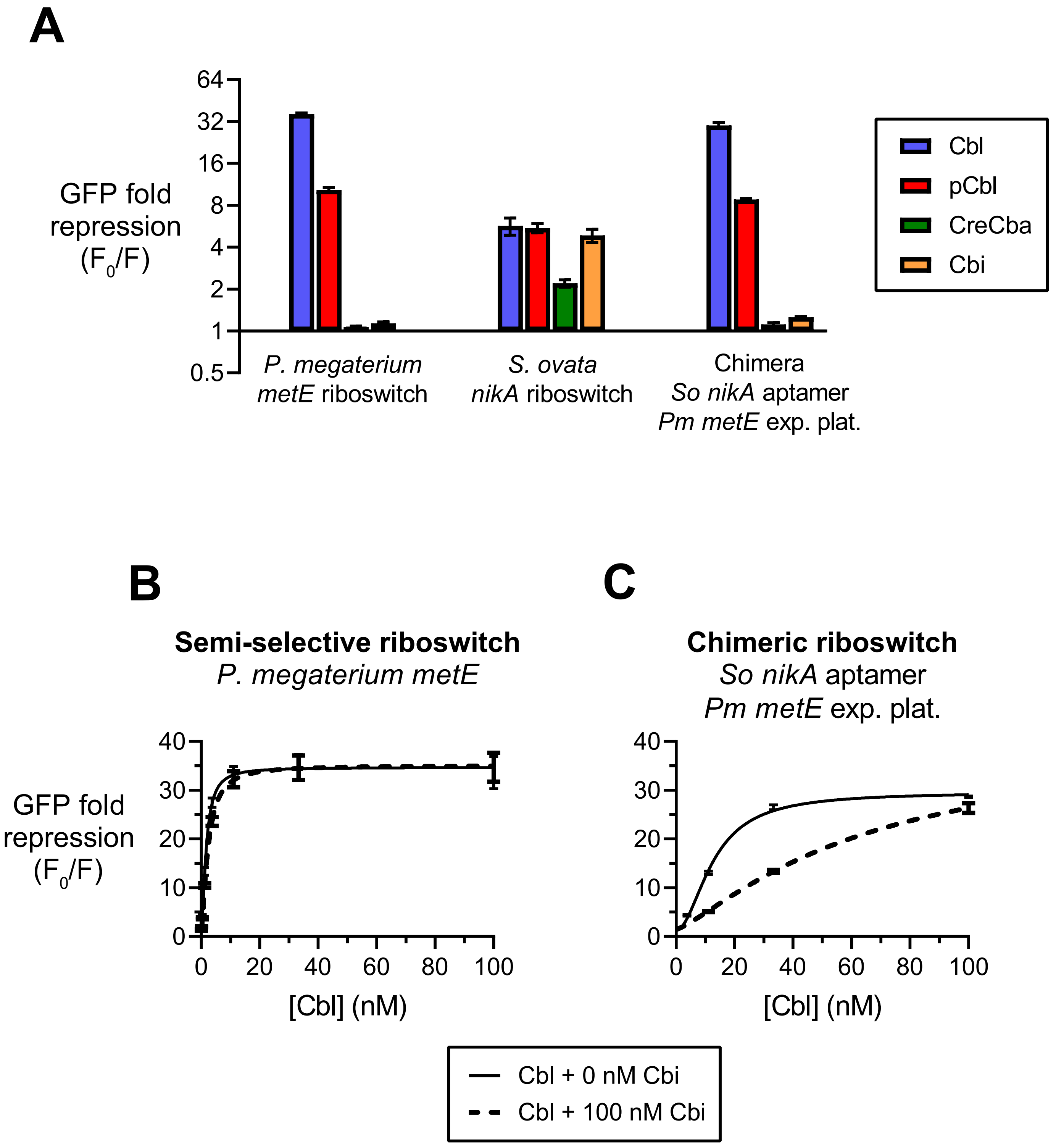
A
GFP fold repression (F_0/F)
64
32
16
8
4
2
1
0.5
P. megaterium metE riboswitch
S. ovata nikA riboswitch
Chimera *So nikA* aptamer *Pm metE* exp. plat.
Cbl
pCbl
CreCba
Cbi
B
Semi-selective riboswitch
P. megaterium metE
C
Chimeric riboswitch
So nikA aptamer
Pm metE exp. plat.
GFP fold repression (F_0/F)
40
30
20
10
0
0
20
40
60
80
100
[Cbl] (nM)
Cbl + 0 nM Cbi
Cbl + 100 nM Cbi
